# Validation of a Disability Assessment Tool Based on the International Classification of Functioning, Disability, and Health in the Chinese Context

**DOI:** 10.3389/fresc.2022.855502

**Published:** 2022-04-25

**Authors:** Jiahui Li, Huaide Qiu, Xia Zhang, Juan Jin, Yuanping Zhao, Juan Yan, Hong Xie, Shouguo Liu, Jianan Li

**Affiliations:** ^1^Center of Rehabilitation Medicine, The First Affiliated Hospital of Nanjing Medical University, Nanjing, China; ^2^XD Group Hospital, Xi'an, China; ^3^School of Nursing, Peking University, Beijing, China; ^4^China Association of Social Security, Beijing, China

**Keywords:** International Classification of Functioning Disability and Health, long-term care, disability assessment, insurance, validation

## Abstract

**Background:**

The common standards of disability assessment for long-term care (LTC) insurance are currently absent. The International Classification of Functioning, Disability and Health (ICF) was designed for a better description of health and functioning, which could fill the demand gap for the standards of disability assessment and be a promising tool for the development of LTC insurance system.

**Objectives:**

To validate a disability assessment scale for disabled elderly individuals based on the ICF for LTC in the Chinese context.

**Methods:**

The present study is a cross-sectional study. A disability assessment tool based on the ICF was developed by referring to other assessment tools and an expert consensus meeting in the initial phase of the study. The developed tool was used to evaluate 1,610 elderly individuals in the LTC institutions. The Cronbach's α coefficient and split-half reliability were applied to test the internal consistency of the tool, while the Interclass correlation coefficients (ICCs) were used to evaluate the interrater reliability (IRR). Factor analysis was performed to verify the construct validity of the tool. The scores from the Medical Outcomes Short Form-12 (SF-12) were correlated with that from the disability assessment tool, to assess the criterion-related validity.

**Results:**

The Cronbach's α coefficient and split-half reliability of the disability assessment tool were 0.969 and 0.877, respectively. The ICCs of the sum scale was 0.85, and the ICCs of each of the 20 items in the scale ranged from 0.78 to 0.94. The items were divided into three factors through analysis, which is consistent with the structure expectation. The scores of each item and the sum score of the disability assessment scale were negatively correlated with the scores of the physical and psychological fields in SF-12 (*p* < 0.001). Overall, the data indicated that the tool was characterized by good internal consistency, IRR, construct validity, and criterion-related validity.

**Conclusions:**

The disability assessment tool based on the ICF is a reliable and valid tool for the collection of information on functioning across various LTC settings. The information of disability provided evidence for the distribution of LTC service and guided the development of LTC insurance standards.

## Introduction

The increasing number of the elderly population and the prevalence of chronic diseases have translated into a public health issue (1–3). As a country with the most elderly population worldwide, China is burdened by elderly with disabilities owing to demographic and health shifts (4–6). By the end of 2018, individuals who are ≥65 years of age accounted for 11.94% of China's population and those aged ≥ 60 years accounted for 17.88% of the population, which have continued to increase over the past 30 years (7). “The fourth China urban and rural living conditions sample survey results” showed that 18.3% of the elderly were either in a disabled or semi-disabled state based on the survey of the daily living activities and care services, and the total number of these elderly individuals was about 40.63 million (8). In this case, the demand for responsive and quality long-term care (LTC), which means health and social service provided by professionals in specific institutions, community and home for those with physical or mental disability, has risen rapidly. In response to this serious problem, China began to implement LTC insurance in 15 cities in 2016 (9). In contrast to healthcare insurance, LTC insurance focuses on nursing expenses incurred by disability individuals, which is interwoven with daily life (10). At present, the LTC insurance system is still not fully established. These LTC insurance piloted cities issued relevant policies, respectively. In some cities, reimbursement amount or rates for LTC insurance are fixed. But in other cities, LTC service levels and the corresponding reimbursement amount were determined by disability assessment results. As LTC is driven by cost and reimbursement, the measurement of an individual's disability with consensus is of great significance for resource allocation. However, there are no common standards of disability assessment for LTC insurance at present. Therefore, as the basis of the LTC insurance system, it is necessary to first set up an evaluation tool to comprehensively assess the health status and ability of the elderly.

The International Classification of Functioning, Disability and Health (ICF) is a classification system issued by the World Health Organization (WHO) aimed to provide a unified standard, language, and framework to describe the health and functioning of an individual (11, 12). The ICF emphasizes that functioning attributes should be given to the interaction of the body functions and structures, activities, and participation with the environmental and personal factors. This concept portrays human disability and health as an integration of multi-dimensional functioning. According to previous studies, the ICF is suitable for application in routine clinical practice as a functional assessment tool with good feasibility, reliability, and validity, which is independent of the health conditions or diseases (13–16). Hence, the ICF could fill the demand gap for the standards of disability assessment, and be a promising tool for the development of the LTC insurance system on guiding LTC service plan and insurance reimbursement. However, the application of the ICF in the assessment of disability is still limited. This is partly attributed to the lack of appropriate tools, as some existing ICF sets are not completely consistent with the demands of the disability assessment for the need of LTC. Currently, the application of the ICF primarily comprises the ICF Generic Set, ICF Rehabilitation Set, and ICF Core Sets for particular diseases. The ICF Generic-6, or ICF Generic-7, represents the minimal information of functioning that should be collected (16). But the information collected may not be enough for the LTC disability assessment. The assessment should be more detailed to refer to disability status discrimination and care service. For the ICF Rehabilitation Set and ICF Core Sets for particular diseases, some categories do not fully match the demands of the disability assessment for LTC, such as d660 assisting others, d770 intimate relationships, and d850 remunerative employment. These categories are not particularly relevant for measuring whether an elder needs to be cared for by others and the related care levels. To facilitate the disability assessment for LTC, it is imperative to develop a new ICF-based tool.

This study aimed to develop and validate a disability assessment tool based on the ICF in the daily routine practice of LTC setting in the Chinese context, and to inform the standards of disability assessment for LTC. The information on the disability of elderly individuals in China provided evidence for the distribution of LTC service levels and contents and guided the development of LTC insurance standards.

## Methods

### Design

In this cross-sectional observational study, elderly individuals residing in 15 insurance-designated LTC institutions (including 14 nursing institution sections and one community homecare section) in China were surveyed from April 2018 to May 2018. To ensure an even distribution of institutions in each region, 15 insurance-designated LTC institutions were selected from eight different cities throughout China. Stratified sampling was used to select elderly individuals from the self-care and non-self-care sections in each institution. The participants were provided with detailed study information, and they gave informed consent to participate in the study. This study followed the Strengthening The Reporting of Observational Studies in Epidemiology (STROBE) guideline (Supplementary Material 1: The STROBE checklist).

### Ethics Approval and Consent to Participate

This study was approved by the Ethics Committee of the First Affiliated Hospital of Nanjing Medical University (No.: 2018-SR-099). All the participants provided informed consent.

### Participants

The inclusion criteria were as follows: (1) elderly in nursing institutions or community homecare aged ≥ 60 years; (2) elderly who provided informed consent. In this step, the disability status of the elderly was not set as one of the inclusion criteria. As this tool was constructed primarily to discriminate the disability of the elderly, the participants of the validation study should be all elderly. The exclusion criteria were the inability to complete the investigation because of sudden diseases or personal reasons. A total of 1,699 elderly individuals were given questionnaires, and 1,610 complete questionnaires were returned. Of these, 103 elderly individuals were assessed by two independent investigators at each time point to assess the interrater reliability (IRR).

### Survey Instrument

In this study, the ICF was used to develop the disability assessment tool through a literature review and an expert consensus meeting. Firstly, the members of the study team collected and arranged the domestic and foreign scales for the assessment of the disability using a literature review to initially identify the concerned domains. Considering the contents and the actual use of the scales, the study referred to the functional domains of the WHO World Health Survey (17), Functional Independence Measurement (FIM) (18), ICF Generic Set and Rehabilitation Set (19), and Chinese disabled classification (20), which have been applied in disability assessment in some cases. In Chinese disabled classification, disabilities are classified into vision, hearing, speech, intellectual, physical, mental and multiple disabilities. Secondly, an expert consensus meeting, which involved experts from different institutions in rehabilitation, nursing, insurance, and other areas, was conducted to identify the functional domains involved in this tool, and then, further determine the ICF categories according to the concerned functional domains to finally make up the ICF-based disability assessment scale. To select the categories efficiently, some common ICF sets with similar functional domains were used for comparison. The background of the tool development was informed to the experts before the meeting. Besides, the experts were also informed of the following principles for the construction of the disability assessment tool: comprehensive assessment of disability, concise contents, and good ability to discriminate among the elderly. Then, discussion and selection through Delphi processes were carried out by the experts until consensus was reached. In detail, after three-round of vote on whether the domains should be involved in the disability assessment tool by hand raise, domains with support ≥75% were included. The same method was then applied to ICF categories selection.

Finally, the developed ICF-based disability assessment tool involved the following eight functional domains: mobility function, self-care ability function, sleep and mental function, emotional function, the sensation of pain function, interpersonal communication and social function, cognitive function, and sensory function. Further, 20 ICF categories were selected to be included in the disability assessment tool by comparing the eight functional domains with the ICF Rehabilitation set, brief ICF Core set for neurological conditions for post-acute care, brief ICF Core set for musculoskeletal conditions for post-acute care, brief ICF core set for cardiopulmonary conditions for post-acute care and brief ICF core set for geriatric patients for post-acute care (21). Among these 20 selected categories, four categories were related to subjective feelings and 16 categories were related to task performance (Table 1).

**Table 1:** The selected 20 ICF categories.

**Classification**		**Category**
Related to task performance	b455	Exercise tolerance functions
	d450	Walking
	d455	Moving around
	b525	Defecation functions
	b620	Urination functions
	d230	Carrying out daily routine
	d510	Washing oneself
	d520	Caring for body parts
	d530	Toileting
	d540	Dressing
	d550	Eating
	d710	Basic interpersonal interactions
	b210	Seeing functions
	b230	Hearing functions
	b144	Memory functions
	b114	Orientation functions
Related to subjective feelings	b130	Energy and drive functions
	b134	Sleep functions
	b152	Emotional functions
	b280	Sensation of pain

**Figure 1:**
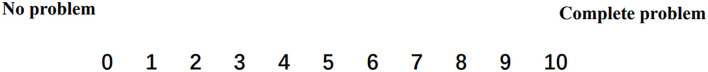
Numeric rating scale (NRS).

The Numerical Rating Scale (NRS) that ranged from 0 (no problem) to 10 (complete problem) was used to evaluate the functioning of the elderly individuals (Figure 1). For the ICF categories related to individuals' subjective feelings, such as b152 Emotional functions and b280 Sensation of pain, self-report measures were taken as a source of information for assessing these categories. The assessors could ask the elderly directly about their feelings based on the description of the category and choose a number between 0 and 10 to represent the functioning of the category. For those who could not follow or answer the questionnaire, these categories were treated as “not applicable” and reasons should be noted, such as consciousness disorders, cognitive impairment or others. Other categories are associated with individuals' performance on the tasks of the categories, such as d450 Walking and d455 Moving around. To ensure consistency of the evaluation in practice for these categories, the researchers divided the continuous score value of 0–10 into 5 levels (Table 2) and designed specific evaluation rules for each level. The details of the assessment for each category are presented in Supplementary Material 2. The assessors scored the elderly according to their task performance and evaluation rules.

**Table 2:** The NRS of disability.

**Level of disability**	**NRS score**
No problem (complete independence)	0
Mild problem (device or supervision)	1
	2
	3
Moderate problem (a small amount of assistance)	4
	5
	6
Severe problem (a large amount of assistance)	7
	8
	9
Complete problem (complete dependence)	10

Short Form-12 (SF-12) was administered to evaluate health and functioning. SF-12 is an abbreviated version of the SF-36, which is extensively applied in international studies to assess quality of life (QOL) domains (22). Due to its characteristics of time-saving operation and recognized QOL evaluation, this study used it for criterion-related validity.

### Data Collection

A manual of how to apply the ICF disability assessment scale in the LTC institutions was developed. The assessment was undertaken by the nurses from each institution. A total of 60 nurses (4 nurses from each institution) received face-to-face training for 1 day before the start of this study. The training content included an introduction to ICF model and classification, the development of the disability assessment tool, what to assess, and how to apply the disability assessment tool. An assessment practice was conducted after the theoretical training, and the questions arose in the assessment practice were explained. The trained nurses conducted questionnaire surveys among the elderly individuals, and the data were collected using an online app. The nurses recorded basic information, ICF assessment and SF-12 of the assessed elderly in the online app, and researchers could view the results through the app background management system. For participants who were unable to finish the questionnaire because of poor listening, language barriers, or other problems, the caregivers helped to provide the functioning information. The questionnaires were collected on the spot, and incomplete questionnaires were rejected.

### Data Analysis

The data were analyzed with the Statistical Package for the Social Sciences (SPSS 20.0). The mean and standard deviation (SD) values were used to describe the category score and sum score. The Cronbach's α coefficient and split-half reliability were applied to test the internal consistency of the scale. The Intraclass correlation coefficients (ICCs) were employed to evaluate the IRR of the ICF disability assessment scale (23). According to the ICCs, the IRR was classified as poor (ICCs <0.40), fair (ICCs 0.40–0.59), good (ICCs 0.60–0.74) and excellent (ICCs > 0.75) (24). The construct validity was assessed using the exploratory factor analysis (EFA), utilizing the principal-axis factoring with the Direct Oblimin Rotation method. The Kaiser-Meyer-Olkin (KMO) test and Bartlett's test of sphericity were performed to identify the adequacy of the study sample and the model fit. The number of factors was determined by the eigenvalues of >1 and scree plot, and the categories with factor loading >0.40 were considered appropriate for loading on a factor (25). The SF-12 scores were calculated in a standardized manner, and the scores in the physical and psychological domain were correlated with the scores of each category and sum scores of the disability assessment scale by correlation analysis to assess the criterion-related validity.

## Results

### Sociodemographic Statistics

A total of 1,699 elderly individuals received the questionnaires, and 1,610 complete questionnaires were collected. The response rate was 94.76%. The assessment was carried out from 1,610 elderly individuals. Their ages ranged from 60 to 105 years, with a mean age of 81.5 (58.71) years; 37.9% were males (*n* = 610) and 62.1% were females (*n* = 1,000).

### Internal Consistency

The Cronbach's α coefficient and split-half reliability of the disability assessment scale were 0.969 and 0.877, respectively, indicating that the scale had good internal consistency.

### Interrater Reliability

The ICCs for the 20 categories and the overall scores of the ICF disability assessment scale are shown in Table 3. The ICCs of the sum score of the scale was 0.85, and the ICCs of the 20 categories in the scale ranged from 0.78 to 0.94, indicating that the IRR of the scale was good (Table 3).

**Table 3:** IRR of disability assessing tool based on ICF (*n* = 103).

**Item**	**ICCs**	**95% CI**			***P*-value**
b455	Exercise tolerance functions	0.92	0.892	0.943	<0.01
d450	Walking	0.78	0.709	0.839	<0.01
d455	Moving around	0.93	0.905	0.950	<0.01
b525	Defecation functions	0.88	0.837	0.913	<0.01
b620	Urination functions	0.88	0.838	0.913	<0.01
d230	Carrying out daily routine	0.87	0.830	0.909	<0.01
d510	Washing oneself	0.86	0.813	0.899	<0.01
d520	Caring for body parts	0.87	0.822	0.904	<0.01
d530	Toileting	0.87	0.829	0.908	<0.01
d540	Dressing	0.90	0.864	0.928	<0.01
d550	Eating	0.88	0.839	0.913	<0.01
b130	Energy and drive functions	0.91	0.875	0.934	<0.01
b134	Sleep functions	0.86	0.815	0.902	<0.01
b152	Emotional functions	0.83	0.776	0.879	<0.01
b280	Sensation of pain	0.87	0.832	0.910	<0.01
b114	Orientation functions	0.82	0.766	0.872	<0.01
b144	Memory functions	0.93	0.902	0.948	<0.01
d710	Basic interpersonal interactions	0.93	0.901	0.948	<0.01
b210	Seeing functions	0.94	0.917	0.956	<0.01
b230	Hearing functions	0.81	0.753	0.865	<0.01
Sum score	0.85	0.801	0.892	<0.01

### Construct Validity

**Figure 2:**
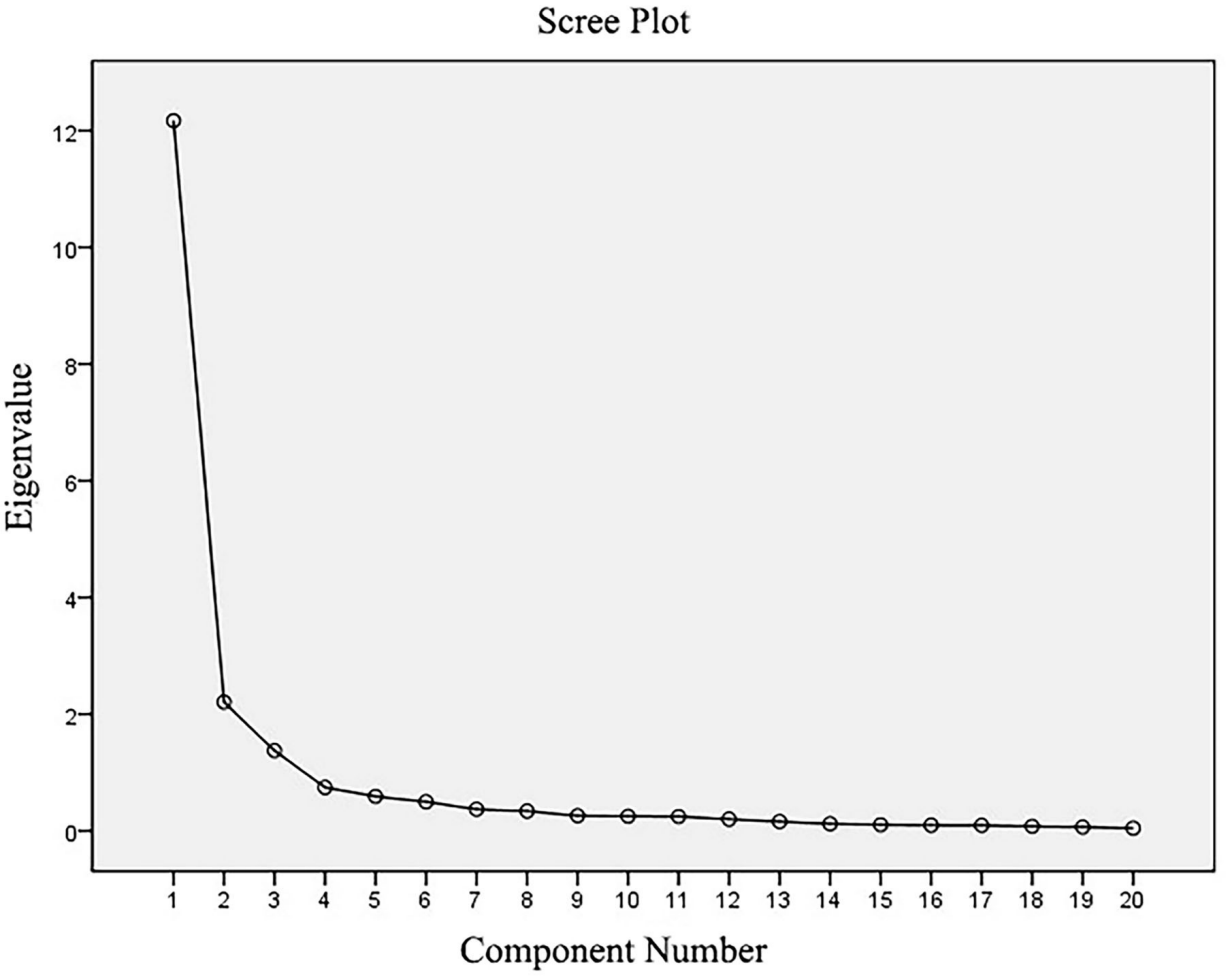
Scree plot from the factor analysis of the disability assessment tool based on the ICF.

The EFA was performed using 20 categories contained in the scale of disability assessment tool as analysis indicators. The results showed that the KMO value was 0.954, and the value of Barlett's test of sphericity was 40839.19 with a significant level (*p* < 0.001), indicating that it was appropriate for the EFA. Using the principal-axis factoring with Direct Oblimin Rotation method, three factors were identified with eigenvalues of >1, and the three factors accounted for 74.368% of the total variance. The extraction of the factors was based on the visual interpretation of the scree plot (Figure 2). The scree plot presented a sharp drop after the third factor, which suggested extracting the three factors. According to the results of the EFA, one category (d550 Eating) had cross-loading on two factors. No category was deleted because of adequate loading on the three factors. The detailed results of the factor analysis are shown in Table 4.

**Table 4:** Factors, items and factor loadings of disability assessing tool based on ICF (*n* = 1,610).

**Items**	**Factor loadings**			**Intercommunity**
	**Factor 1**	**Factor 2**	**Factor 3**	
d450 Walking	0.977	−0.013	−0.06	0.865
d510 Washing oneself	0.968	0.017	−0.043	0.890
d520 Caring for body parts	0.963	0.010	−0.029	0.894
d530 Toileting	0.931	0.017	0.017	0.902
d230 Carrying out daily routine	0.924	0.026	0.004	0.878
d455 Moving around	0.919	−0.006	0.003	0.844
d540 Dressing	0.913	0.012	0.029	0.882
b455 Exercise tolerance functions	0.857	0.047	−0.017	0.747
b620 Urination functions	0.609	−0.003	0.316	0.743
b525 Defecation functions	0.590	0.001	0.320	0.722
d550 Eating	0.462	0.052	0.418	0.704
b134 Sleep functions	−0.050	0.821	−0.038	0.623
b152 Emotional functions	0.015	0.751	0.141	0.686
b130 Energy and drive functions	0.136	0.739	0.046	0.682
b280 Sensation of pain	−0.017	0.712	−0.056	0.469
d710 Basic interpersonal interactions	0.086	0.038	0.833	0.836
b144 Memory functions	0.123	0.009	0.790	0.786
b210 Seeing functions	−0.108	0.058	0.751	0.495
b114 Orientation functions	0.174	−0.003	0.742	0.763
b230 Hearing functions	−0.009	−0.009	0.690	0.462

*The gray marker factor loading >0.40*.

### Criterion-Related Validity

The scores of the physical and psychological domains of SF-12 were calculated. The correlation analysis of SF-12 with the scores of each category and sum scores of the ICF disability assessment scale, which generated by summing up all the scores of 20 categories, are shown in Table 5.

**Table 5:** Criterion-related validity of ICF disability assessing scale (*n* = 1,610).

**Items**		**Physiological function**		**Psychological function**	
		***r***	***P***	***r***	***P***
	Sum scores	−0.596	<0.001	−0.332	<0.001
b455	Exercise tolerance functions	−0.608	<0.001	−0.278	<0.001
d450	Walking	−0.593	<0.001	−0.260	<0.001
d455	Moving around	−0.578	<0.001	−0.260	<0.001
b525	Defecation functions	−0.485	<0.001	−0.264	<0.001
b620	Urination functions	−0.475	<0.001	−0.259	<0.001
d230	Carrying out daily routine	−0.599	<0.001	−0.261	<0.001
d510	Washing oneself	−0.600	<0.001	−0.268	<0.001
d520	Caring for body parts	−0.611	<0.001	−0.265	<0.001
d530	Toileting	−0.587	<0.001	−0.275	<0.001
d540	Dressing	−0.592	<0.001	−0.262	<0.001
d550	Eating	−0.461	<0.001	−0.273	<0.001
b130	Energy and drive functions	−0.308	<0.001	−0.332	<0.001
b134	Sleep functions	−0.167	<0.001	−0.393	<0.001
b152	Emotional functions	−0.254	<0.001	−0.348	<0.001
b280	Sensation of pain	−0.277	<0.001	−0.441	<0.001
b114	Orientation functions	−0.396	<0.001	−0.245	<0.001
b144	Memory functions	−0.414	<0.001	−0.286	<0.001
d710	Basic interpersonal interactions	−0.426	<0.001	−0.316	<0.001
b210	Seeing functions	−0.332	<0.001	−0.229	<0.001
b230	Hearing functions	−0.329	<0.001	−0.303	<0.001

The results showed that the scores of each category and sum scores of the ICF disability assessment scale were significantly negatively correlated with the scores of the physical and psychological domains in SF-12 (*p* < 0.001). The categories in the disability assessment scale that reflect the self-care level, such as activity endurance, caring for one's body parts, washing oneself, and carrying out a daily routine, had a higher correlation with the SF-12 physical function domain than with the other categories. The categories such as pain sensation function, sleep function, and emotional function were more correlated with the psychological function domain than with the other categories.

## Discussion

In the early stages of this study, a disability assessment tool based on the ICF was developed by referring to other assessment tools and expert consensus. This study validated the developed disability assessment tool of the ICF in the daily routine practice in the Chinese context and aimed to provide references for the standards of disability assessment for LTC. The results from 1,610 elderly individuals showed that the tool had good internal consistency, IRR, construct validity, and criterion-related validity.

In this study, the Cronbach's α coefficient and split-half reliability of the disability assessment scale were 0.969 and 0.877, respectively, indicating that the design of the tool was reasonable and each category can reflect the degree of disability consistently (26). As compared with other similar tools, the disability assessment tool based on the ICF demonstrated better internal consistency (27).

Previous studies showed that IRR may be influenced by the ICF categories description, assessment methods, professional background, and experience of the assessors (28–30). A study about the clinical application of the ICF Generic Set suggested that there were differences in the understanding of the categories among the evaluators in China, and the assessors could not well distinguish the boundaries between the different generic ICF qualifier ratings (five response options) (15). Additionally, some studies without the standard guidance of qualifier ratings usually resulted in low reliability (30, 31). According to a large multicenter cohort study among 20 provinces in China, the scale used in combination with the ICF Generic Set and the 0–10 NRS, an instrument that every assessor in China is familiar with, has good IRR and intra-rater reliability (16). In this study, we adopted 0–10 NRS, developed evaluation criteria, and trained assessors prior to the implementation of the assessment. In addition, the NRS evaluation criterion for each category was shown in the app for the evaluators to understand during the assessment. Finally, it was found that the IRR of the scale was good. Some categories, such as d450 Walking, b152 Emotional functions, b114 Orientation functions, and b230 Hearing functions, achieved somewhat lower ICCs. However, when compared with other studies, the values of the ICCs were acceptable for daily routine practice (16, 28). For these categories, relevant descriptions can be improved and researchers can place additional focus on them during training to increase the reliability.

Considering that the categories in the scale were correlated, the principal-axis factoring was adopted with Direct Oblimin Rotation method in the EFA to analyze the construct validity. The results suggested that this scale is a three-factor structure, which is in line with the expectation for properties of a disability assessment tool. Factor 1 was named self-care ability and activity, factor 2 was named emotion and spirit, and factor 3 was named cognition and perception. At an acceptable level of loading ≥0.4, each category is assigned to a factor, except d550 Eating. The factor loadings of the category d550 Eating on factor 1 and factor 3 were >0.4, implying that there is cross-loading. After the theoretical analysis, the content attribute of category d550 Eating was inclined to self-care ability more than toward cognition and perception, so it was finally categorized into factor 1.

Therefore, using the EFA, the 20 ICF categories were divided into three dimensions: self-care ability and activity (eleven categories: d450 Walking, d510 Washing oneself, d520 Caring for body parts, d530 Toileting, d230 Carrying out a daily routine, d455 Moving around, d540 Dressing, b455 Exercise tolerance functions, b620 Urination functions, b525 Defecation functions and d550 Eating), emotion and spirit (four categories: b134 Sleep functions, b152 Emotional functions, b130 Energy and drive functions and b280 Sensation of pain) and cognition and perception (five categories: d710 Basic interpersonal interactions, b144 Memory functions, b210 Seeing functions, b114 Orientation functions and b230 Hearing functions). These results were consistent with the previous findings on the Internal Dimensional Consistency Analysis of the disability assessment tool, which means that the tool conformed to the expected questionnaire structure.

The conventional disability assessment tools, such as the Barthel index or the Functional Independence index, do not cover all the functioning domains (32). Instead of focusing only on self-care ability, the developed ICF-based disability assessment tool in this study fully evaluates the individual-related functioning from three dimensions. Compared with another questionnaire about disability, the World Health Organization Quality of Life-BREF (WHOQOL-BREF) questionnaire, the disability assessment tool based on the ICF does not include an environmental dimension, which made a large contribution to the overall quality of life (33). Considering that this tool would be mainly used in LTC insurance institutions, some environmental factors may play limited roles in the disability of the individuals living in institutions, especially those with severe disability and under plenty of care. However, there are some other environmental factors, such as service systems, policies, family relationships and others, associated with disability status. The necessity of these factors for the LTC insurance disability assessment tool merits further discussion in future research.

Previous studies on the ICF used SF-36 to test the criterion-related validity (34, 35). Because of the large sample size of this study, SF-12, the simplified version of SF-36, was used to analyze the criterion-related validity of the assessment tool. The results of the present study showed that the scores of each category and sum scores of the disability assessment scale were significantly negatively correlated with the scores of the physical and psychological fields in SF-12, which indicated that the disability assessment tool was consistent with measurements' characteristics of SF-12. The categories reflecting self-care ability in the disability assessment tool, such as washing oneself, caring for body parts, carrying out daily routine and exercise tolerance functions, had more correlation with the physical domain in SF-12. On the other hand, the categories about emotion, such as the sensation of pain, sleep functions and emotional functions, revealed more correlation with the psychological domain in SF-12. Some subjective categories in the ICF did not correlate well with SF-36 or SF-12 in other studies (16, 35). However, this phenomenon was not found in the present study.

Wynia et al. (36) used a measurement tool based on the ICF to assess individuals with multiple sclerosis and examined the relative validity of the tool. It was found that the tool based on the ICF performed equally or slightly worse than the professional measures in specific domains, but better than the multidimensional health measures such as SF-36. For a comprehensive and multidimensional assessment of health and disability situations, the tool based on the ICF is a good choice.

There are some strengths in the present study. Although tools based on the ICF have been developed in various fields, there is a lack of standard tools for the assessment of disability for LTC insurance. Through an expert consensus meeting, the current study developed a disability assessment tool based on the ICF for LTC insurance that could evaluate individuals' disability comprehensively. In the validation of the tool, individuals from 15 insurance-designated LTC institutions in different regions of China were surveyed to eliminate the regional differences. This tool could fill blank for the assessment of disability for LTC insurance. Information gathered from this assessment tool can contribute to the grading of disability, care service and insurance payment.

The main limitation of this study is that only one community homecare section was surveyed among 15 insurance-designated LTC institutions in the validation, so it is not easy to generalize this assessment tool to the whole community homecare individuals. A low number of individuals and multiple assessments from one evaluator may lead to relatively unreliable results in the IRR analysis. The methods adopted in this study were easy to understand for people with no medical background such as the members of the insurance companies, but are likely to be oversimplified compared with the latest methods and theories. Therefore, future studies including a large number of individuals and evaluators and with more rigorous methods are required for more accurate and reliable results. Besides, the primary objective of the study was to provide references for LTC insurance. One of the most important abilities of any assessment tool is its ability to discriminate elderly individuals' disability statuses and levels. This was not determined in the present study. The construction and validation of the assessment tool is a preliminary stage for the whole project, and further studies are planned to identify the discrimination ability of this tool.

## Conclusions

In this study, the disability assessment tool based on the ICF with good internal consistency, IRR, construct validity, and criterion-related validity was developed. According to the overall analysis, this tool can effectively and comprehensively assess the disability of the elderly. Therefore, it can be recommended for routine use in the disability assessment of LTC. Further data and analysis are needed to assess its applicability in insurance.

## Data Availability Statement

The raw data supporting the conclusions of this article will be made available by the authors, without undue reservation.

## Ethics Statement

The studies involving human participants were reviewed and approved by the Ethics Committee of the First Affiliated Hospital of Nanjing Medical University. Written informed consent for participation was not required for this study in accordance with the national legislation and the institutional requirements.

## Author Contributions

JHL wrote the first draft of this manuscript and contributed to the interpretation of data. HQ consulted on data analysis and was a major contributor in writing the manuscript. XZ, JJ, and YZ coordinated data collection and revised the manuscript for critical content. JY designed the study and revised the manuscript for critical content. JNL consulted on data analysis, designed and supervised the study, and revised the manuscript for critical content. SL designed the study, analyzed the data, and revised the manuscript for critical content. HX designed and supervised the study, and revised the manuscript for critical content. All authors read and approved the final manuscript.

## Funding

This study was funded by the Nanjing Municipal Science and Technology Bureau (Grant no. 2019060002). The funding bodies had no role in the study design, data collection, analysis, and interpretation of data.

## Conflict of Interest

The authors declare that the research was conducted in the absence of any commercial or financial relationships that could be construed as a potential conflict of interest.

## Publisher's Note

All claims expressed in this article are solely those of the authors and do not necessarily represent those of their affiliated organizations, or those of the publisher, the editors and the reviewers. Any product that may be evaluated in this article, or claim that may be made by its manufacturer, is not guaranteed or endorsed by the publisher.
