# Supplementary material for: Validation of a Disability Assessment Tool Based on the International Classification of Functioning, Disability, and Health in the Chinese Context
Source: Front Rehabil Sci. 2022 Apr 25;3:855502. doi: 10.3389/fresc.2022.855502 (PMC9397936; doi:10.3389/fresc.2022.855502)
Supplement: Supplementary file 2 [file Table_2.DOCX]

Supplementary S2. The disability assessing tool based on ICF in English and Chinese

|  | item | content |
| --- | --- | --- |
| b455 | Exercise tolerance functions  运动耐受功能 | Description: functions related to respiratory and cardiovascular capacity as required for enduring physical exertion.  Simple description: ability to perform exercise continuously for some level of duration and at definite intensity.  描述：与呼吸和心血管能力有关的适应持续体力消耗的功能。  简洁描述：能够持续进行一定时长和强度的运动能力。 |
|  |  | Inclusions: functions of physical endurance, aerobic capacity, stamina and fatiguability  包括：身体耐力，有氧耐受力、抵抗力和易疲劳性的功能 |
|  |  | Exclusions: functions of the cardiovascular system (b410-b429); haematological system functions (b430); respiration functions (b440); respiratory muscle functions (b445); additional respiratory functions (b450)  不包括：心血管系统功能（b410-429）；血液系统功能（b430）；呼吸功能（b440）；呼吸肌功能（b445）；辅助呼吸功能（b450） |
|  |  | NRS assessment:  0: no fatigue in doing daily activities 1-3: feel fatigued in doing daily activities occasionally 4-6: feel fatigued in doing daily activities frequently  7-9: feel fatigued in doing daily activities most of the time  10: cannot complete daily activities  NRS评估：  0分：正常完成日常生活活动，无疲劳，抵抗力强  1-3分：可正常完成日常事物，有疲劳感，抵抗力一般  4-6分：可完成日常事物，经常疲劳，抵抗力较差  7-9分：日常事物完成费力，绝大多数时候都很疲劳，抵抗力差  10分：不能完成日常事物，极易疲劳，抵抗力极差 |
| d450 | Walking  步行 | Description: moving along a surface on foot, step by step, so that one foot is always on the ground, such as when strolling, sauntering, walking forwards, backwards, or sideways.  Simple description: moving along a surface on foot and one foot is always on the ground.  描述：靠脚在地面一步步走动，总是一只脚在地面，如漫步、踱步、向前、后或两侧行走。  简洁描述：用脚在地面上移动，总有一只脚在地面 |
|  |  | Inclusions: walking short or long distances; walking on different surfaces; walking around obstacles  包括：短距离或长距离步行；不同地面步行；绕障碍步行 |
|  |  | Exclusions: transferring oneself (d420); moving around (d455)  不包括：移动自身（d420），到处移动（d455） |
|  |  | NRS assessment:  0: can neighborhood walk independently without a risk of falling  1-3: have a risk of falling in walking and need supervision from others  4-6: need a small amount of support from others while walking  7-9: need a large amount of support from others while short distance walking (indoor walking)  10: be unable to walk and completely dependent on others  NRS评估：  0分：个体能够独立社区步行，无摔倒风险  1-3分：个体在步行过程中，存在摔倒风险，需要他人监控  4-6分：个体在步行过程中需要他人小量的扶持帮助  7-9分：个体在他人大量扶持帮助下能进行短距离步行（室内步行）  10分：无法步行，完全依赖 |
| d455 | Moving around  到处移动 | Description: moving the whole body from one place to another by means other than walking, such as climbing over a rock or running down a street, skipping, scampering, jumping, somersaulting or running around obstacles.  Simple description: moving from one place to another by means other than walking, such as running, jumping or climbing over a rock.  描述：移动身体从一个至另一个位置，通过除步行以外的各种方式，如：攀岩，或者穿过街道、蹦、奔跑、跳跃、绕障碍跑。  简洁描述：以步行以外的方式，从一地移动到另一地，如跑、跳、攀岩等。 |
|  |  | Inclusions: crawling, climbing, running, jogging, jumping, and swimming  包括：爬行，攀登，奔跑，慢跑，跳跃和游泳 |
|  |  | Exclusions: transferring oneself (d420); walking (d450)  不包括：移动自身（d420），步行（d450） |
|  |  | NRS assessment:  0: can move around independently  1-3: need supervision from others while moving around  4-6: need a small amount of support from others while moving around  7-9: need a large amount of support from others while moving around  10: be unable to move around and completely dependent on others  NRS评估：  0分：个体能够独立的穿过街道、上下楼梯，或使用轮椅到处移动  1-3分：个体需要在监护下穿过街道、上下楼梯，或使用轮椅需要指示帮助  4-6分：个体在穿过街道、上下楼梯或轮椅使用时需要小量接触式帮助  7-9分：个体在穿过街道、上下楼梯或轮椅使用时需要大量接触式帮助  10分：个体需要完全依赖他人进行到处移动 |
| b525 | Defecation functions  排便 | Description: functions of elimination of wastes and undigested food as faeces and related functions.  描述：以粪便形式将废弃物和未消化食物排除体外的功能及有关功能。 |
|  |  | Inclusions: functions of elimination, faecal consistency, frequency of defecation; faecal continence, flatulence; impairments such as constipation, diarrhoea, watery stool and anal sphincter incompetence or incontinence  包括：排便、大便稠度、排便次数、大便控制、肠胀气的功能；如便秘、腹泻、水样便、括约肌失能或失禁的损伤 |
|  |  | Exclusions: digestive functions (b515); assimilation functions (b520); sensations associated with the digestive system (b535)  不包括：消化功能（b515）；同化功能（b520）；与消化系统相关的感觉（b535） |
|  |  | NRS assessment:  0: can control the elimination of faeces at will all the time  1-3: can control the elimination of faeces at will most of the time, occasionally (less than once a week) have constipation or fecal incontinence  4-6: can control the elimination of faeces at will most of the time, occasionally (more than once a week) have constipation or fecal incontinence  7-9: have constipation or fecal incontinence most of the time  10: have severe constipation or stool incontinence  NRS评估：  0分：可自行控制大便排出，无问题  1-3分：大部分时间可自行控制大便，有时出现（1周以上）便秘或大便失禁  4-6分：大部分时间可自行控制大便，有时出现（1周以内）便秘或大便失禁  7-9分：绝大部分时间均出现便秘或大便失禁  10分：患者严重便秘或者大便失禁 |
| b620 | Urination functions  排尿 | Description: functions of discharge of urine from the urinary bladder.  Simple description: ability to control discharge of urine at will.  描述：尿液从膀胱中排泄出去的功能。  简洁描述：随意控制和排出尿液的能力。 |
|  |  | Inclusions: functions of urination, frequency of urination, urinary continence; impairments such as in stress, urge, reflex, overflow, continuous incontinence, dribbling, automatic bladder, polyuria, urinary retention and urinary urgency  包括：排尿、排尿次数、排尿控制功能；如应激性膀胱、窘迫性膀胱、反射性膀胱、充盈性膀胱、持续性尿失禁、滴尿、自主膀胱、多尿症、尿潴留和尿急的损伤 |
|  |  | Exclusions: urinary excretory functions (b610); sensations associated with urinary functions (b630)  Search Fields  不包括：尿液形成功能（b610）；与泌尿功能相关的感觉（b630） |
|  |  | NRS assessment:  0: normal urination frequency and urination control  1-3: normal during daytime, but increased frequency of urination and poor urine control during nighttime  4-6: normal during most of the daytime and poor urine control during nighttime  7-9: cannot control urine during most of the daytime, uracratia during nighttime  10: uracratia and unable to control urination completely  NRS评估：  0分：可自行控制排尿，排尿次数、小便控制均正常  1-3分：白天可自行控制小便次数，夜间出现排尿次数增多、小便控制较差  4-6分：白天大部分时间可自行控制小便，偶出现尿失禁，夜间控制小便较差  7-9分：白天大部分时间不能控制小便，夜间出现尿失禁  10分：小便失禁，完全不能控制小便 |
| d230 | Carrying out daily routine | Description: carrying out simple or complex and coordinated actions in order to plan, manage and complete the requirements of day-to-day procedures or duties, such as budgeting time and making plans for separate activities throughout the day.  Simple description: planning, managing and completing daily procedures or duties.  描述：为了对日复一日的日常事务作出计划、安排并完成而进行的简单或复杂及协调性的活动，如为整日的各种活动安排时间并作出计划。  简洁描述：计划、安排并完成日常生活事务。 |
|  |  | Inclusions: managing and completing the daily routine; managing one's own activity level  包括：安排和完成日常事务；控制自身活动水平 |
|  |  | Exclusions: undertaking multiple tasks (d220)  不包括：从事多项任务（d220） |
|  |  | NRS assessment:  0: can plan, arrange and complete daily tasks independently without help  1-3: need others’ guidance in carry out daily routine  4-6: need a small number of help in carry out daily routine  7-9: need a large number of help in carry out daily routine  10: completely depend on others in carry out daily routine  NRS评估：  0分：个体能够完全独立计划、安排和完成日常事务，无需帮助  1-3分：个体在计划、安排和完成日常事务需要指示帮助  4-6分：个体在计划、安排和完成日常事务需要小量接触式帮助  7-9分：个体在计划、安排和完成日常事务需要大量接触式帮助  10分：个体完全依赖他人进行日常事务 |
| d510 | Washing oneself | Description: washing and drying one's whole body, or body parts, using water and appropriate cleaning and drying materials or methods, such as bathing, showering, washing hands and feet, face and hair, and drying with a towel.  Simple description: washing and drying one's whole body, or body parts.  描述：用水和适当的清洁及干燥材料或方法盥洗和擦干自己的全身或身体各部，如洗澡、淋浴、洗手、脚、脸和头发以及使用毛巾擦干。  简洁描述：清洁和擦干全身或部分身体。 |
|  |  | Inclusions: washing body parts, the whole body, and drying oneself  包括：盥洗身体各部、全身和擦干身体 |
|  |  | Exclusions: caring for body parts (d520); toileting (d530)  不包括：护理身体各部（d520）；如厕（d530） |
|  |  | NRS assessment:  0: can wash body parts independently without help  1-3: need others’ guidance in washing body parts  4-6: need others’ help in washing body but can complete washing face or feet by oneself  7-9: need others’ help in washing all body parts  10: completely depend on others in washing all body parts  NRS评估：  0分：个体可以独立的完成盥洗全身各部，包括洗澡、洗脸、洗脚等  1-3分：个体盥洗全身各部时需要他人监控或指导  4-6分：个体在洗澡时需要他人帮助，能够自行完成洗脸或洗脚  7-9分：个体在盥洗身体各部时都需要他人帮助  10分：个体在盥洗身体各部时完全依赖他人 |
| d520 | Caring for body parts | Description: looking after those parts of the body, such as skin, face, teeth, scalp, nails and genitals, that require more than washing and drying.  Simple description: looking after skin, teeth, hair, nails and genitals.  描述：护理身体各部位，如皮肤、面部、牙齿、头皮、指甲和生殖器，这些部位不仅需要清洗和擦干，还需要护理。  简洁描述：护理皮肤、牙齿、毛发、指（趾）甲和生殖器等。 |
|  |  | Inclusions: caring for skin, teeth, hair, finger and toe nails, and nose  包括：护理皮肤、牙齿、毛发、指（趾）甲 |
|  |  | Exclusions: washing oneself (d510); toileting (d530)  不包括：盥洗自身（d510）；如厕（d530） |
|  |  | NRS assessment:  0: can care body parts independently without help  1-3: need others’ guidance in caring body parts  4-6: need a small number of contact help in caring body parts  7-9: need a large number of contact help in caring body parts  10: completely depend on others in caring body parts  NRS评估：  0分：个体可以独立的完成身体各部护理  1-3分：个体在进行身体各部护理时需要他人监控或指导  4-6分：个体在护理身体各部时需要他人小量接触式帮助  7-9分：个体在护理身体各部时需要他人大量接触式帮助  10分：个体在护理身体各部时完全依赖他人 |
| d530 | Toileting | Description: planning and carrying out the elimination of human waste (menstruation, urination and defecation), and clean oneself afterwards.  Simple description: completing elimination of human waste in an appropriate way.  描述：安排和完成人体废弃物（月经、小便和大便）的排泄，然后清洁身体。  简洁描述：以恰当的方式完成大小便和经期护理。 |
|  |  | Inclusions: regulating urination, defecation and menstrual care  包括：控制大小便和月经护理 |
|  |  | Exclusions: washing oneself (d510); caring for body parts (d520)  不包括：盥洗自身（d510）；护理身体各部（d520） |
|  |  | NRS assessment:  0: can carry out the elimination of human waste independently and clean oneself afterwards independently without help  1-3: need others’ guidance in toileting  4-6: need a small number of contact help in toileting  7-9: need a large number of contact help in toileting  10: completely depend on others in toileting  NRS评估：  0分：个体可以独立的完成大小便排放，并进行会阴部清洁，不需要他人帮助  1-3分：个体在如厕时需要他人监控或指导  4-6分：个体在如厕时需要他人小量接触式帮助  7-9分：个体在如厕时需要他人大量接触式帮助  10分：个体在如厕时完全依赖他人 |
| d540 | Dressing | Description: carrying out the coordinated actions and tasks of putting on and taking off clothes and footwear in sequence and in keeping with climatic and social conditions, such as by putting on, adjusting and removing shirts, skirts, blouses, pants, undergarments, saris, kimono, tights, hats, gloves, coats, shoes, boots, sandals and slippers.  Simple description: Choosing, putting on and taking off clothes and footwear in keeping with climatic and social conditions.  描述：在与当时的气候和社会情况相一致的前提下，按照先后顺序通过协调性动作完成穿衣、裙子、上衣、裤子、各层内衣、莎丽、和服、紧身衣、帽子、手套、大衣、鞋袜、靴子、凉鞋和拖鞋。  简洁描述：根据气候和环境选择衣物和鞋袜，并以适当的方式穿脱。 |
|  |  | Inclusions: putting on or taking off clothes and footwear and choosing appropriate clothing  包括：穿上或脱下衣服和鞋袜以及选择合适的衣着 |
|  |  | Exclusions: none  不包括：无 |
|  |  | NRS assessment:  0: can independently put on or take off clothes and footwear in an appropriated way  1-3: need others’ guidance in dressing  4-6: need a small number of contact help in dressing  7-9: need a large number of contact help in dressing  10: completely depend on others in dressing  NRS评估：  0分：个体可以独立并以适合的方式的完成穿脱衣服、鞋袜，不需要他人帮助  1-3分：个体在穿脱衣服、鞋袜需要他人监控或指导  4-6分：个体能完成部分衣服、鞋袜的穿脱，如只能穿上衣或裤子，无法完成全部  7-9分：个体在穿脱衣服、鞋袜时需要他人大量帮助  10分：个体在穿脱衣服鞋袜时完全依赖他人 |
| d550 | Eating | Description: carrying out the coordinated tasks and actions of eating food that has been served, bringing it to the mouth and consuming it in culturally acceptable ways, cutting or breaking food into pieces, opening bottles and cans, using eating implements, having meals, feasting or dining.  Simple description: Using appropriate eating implements to bring food to the mouth and consume it  描述：通过协调性动作去吃所提供的食物，将食物送进嘴中并按照有教养的方式吃喝，把食物切开或切成片、开瓶和罐头、使用各种餐具、进餐、出席宴会及午餐。  简洁描述：使用适当的器具将食物送入嘴中并能咽下。 |
|  |  | Inclusions: none  包括：无 |
|  |  | Exclusions: drinking (d560)  不包括：喝（d560） |
|  |  | NRS assessment:  0: can use eating implements to bring food to the mouth and consume it without choke  1-3: need others’ guidance in eating  4-6: can eat independently most of time, or choke occasionally  7-9: need help most of eating time, or choke frequently  10: completely depend on others in eating or unable to consume  NRS评估：  0分：个体可以独立使用餐具将食物送进口中并咽下，没有呛咳  1-3分：个体在进食过程中需要监控或指示  4-6分：个体在大部分时候能独立进食，偶有呛咳  7-9分：个体在进食过程中需要他人接触式帮助，频发呛咳  10分：个体完全依赖他人帮助进食，或吞咽困难 |
| b130 | Energy and drive functions | Description: general mental functions of physiological and psychological mechanisms that cause the individual to move towards satisfy specific needs and general goals in a persistent manner.  Simple description: physical ability and initiative required to satisfy specific needs and general goals.  描述：驱使个体以持久的方式为满足特殊需要和总目标而不懈追求的生理和心理机制的一般精神功能。  简洁描述：为达成一般目标和满足特殊需求而具备的体能和主观能动性。 |
|  |  | Inclusions: functions of energy level, motivation, appetites, craving (including craving for substances that can be abused), and impulse control  包括：能量水平、动机、食欲、成瘾（包括可能导致滥用成瘾物质）以及冲动控制的功能 |
|  |  | Exclusions: consciousness functions (b110); temperament and personality functions (b126); sleep functions (b134); psychomotor functions (b147); emotional functions (b152)  不包括：意识功能（b110）；气质功能（b126）；睡眠功能（b134）；心理运动（b147）；情绪功能（b152） |
|  |  | NRS assessment:  0-10: self-reported subjective experience  NRS评估：  0-10：自我报告的主观体验 |
| b134 | Sleep functions | Description: general mental functions of periodic, reversible and selective physical and mental disengagement from one's immediate environment accompanied by characteristic physiological changes.  Simple description: ability to sleep selectively, maintain the appropriate time and quality to satisfy daily needs.  描述：从个人即时所处的以生理变化为特征的环境中产生周期性、可逆性和选择性身体和心理解脱的一般精神功能。  简洁描述：能选择性地进行睡眠并保持适当的时间和质量，满足日常所需。 |
|  |  | Inclusions: functions of amount of sleeping, and onset, maintenance and quality of sleep; functions involving the sleep cycle, such as in insomnia, hypersomnia and narcolepsy  包括：睡眠量、睡眠开始、睡眠维持和质量的功能，涉及睡眠周期的功能，如失眠、嗜眠症、发作性睡病 |
|  |  | Exclusions: consciousness functions (b110); energy and drive functions (b130); attention functions (b140); psychomotor functions (b147)  不包括：意识功能（b110）；能量和驱力功能（b130）；注意力功能（b140）；心理运动功能（b147） |
|  |  | NRS assessment:  0-10: self-reported subjective experience  NRS评估：  0-10：自我报告的主观体验 |
| b152 | Emotional functions | Description: specific mental functions related to the feeling and affective components of the processes of the mind.  Simple description: ability to produce appropriate feeling and to manage different feeling  描述：与感情和心理活动中的情感成份有关的特殊精神功能。  简洁描述：个体产生恰当的情感并且管理各种不同情感的能力。 |
|  |  | Inclusions: functions of appropriateness of emotion, regulation and range of emotion; affect, sadness, happiness, love, fear, anger, hate, tension, anxiety, joy, sorrow; lability of emotion; flattening of affect  包括：情感的适度性、情感的调节和范围：感情；悲伤、幸福、热爱、恐惧、愤怒、仇恨、紧张、焦虑、快乐、悲哀；情绪的易变性；感情单调的功能 |
|  |  | Exclusions: temperament and personality functions (b126); energy and drive functions (b130)  不包括：气质和人格功能（b126）；能量和驱力功能（b130） |
|  |  | NRS assessment:  0-10: self-reported subjective experience  NRS评估：  0-10：自我报告的主观体验 |
| b280 | Sensation of pain | Description: sensation of unpleasant feeling indicating potential or actual damage to some body structure.  描述：身体某处受到潜在或实际损害而感到不舒服的感觉。 |
|  |  | Inclusions: sensations of generalized or localized pain, in one or more body part, pain in a dermatome, stabbing pain, burning pain, dull pain, aching pain; impairments such as myalgia, analgesia and hyperalgesia  包括：在身体一处或多处的全身性或局部性疼痛、皮肤疼痛、刺疼、灼疼、钝疼、疼痛；如肌疼、痛觉和痛觉过敏的损伤 |
|  |  | Exclusions: none  不包括：无 |
|  |  | NRS assessment:  0-10: self-reported subjective experience  NRS评估：  0-10：自我报告的主观体验 |
| b114 | Orientation functions | Description: general mental functions of knowing and ascertaining one's relation to time, to place, to self, to others, to objects and to space.  描述：知道并确认与自我、他人、时间及周围环境关系的一般精神功能。 |
|  |  | Inclusions: functions of orientation to time, place and person; orientation to self and others; disorientation to time, place and person  包括：时间定向、方位定向和人物定向功能；自我定向和他人定向功能；时间、方位和人物定向障碍 |
|  |  | Exclusions: consciousness functions (b110); attention functions (b140); memory functions (b144)  不包括：意识功能（b110）；注意力功能（b140）；记忆功能（b144） |
|  |  | NRS assessment:  0: accurate time perception; can go far alone and quickly grasp the new environment  1-3: know the year and month but sometimes difference a few days about time perception; can go to and from the near street alone, know the name and location of the present residence, but do not know the way home  4-6: poor time perception, do not know the year, month and day, only know the first or second half of the year;  can only move alone in the vicinity of the home, only know the name of the current residence, but do not know the location  7-9: poor time perception, do not know the year, month and day, only know morning or afternoon; can only move in the vicinity of the home, do not know the name and the location of the current residence  10: have no time perception; cannot go outside alone  NRS评估：  0分：时间观念（年、月、日、时）清楚；可单独出远门，能很快掌握新环境的方位  1-3分：时间观念有些下降，年、月、日清楚，但有时相差几天；可单独来往于近街，知道现住地的名称和方位，但不知回家路线  4-6分：时间观念较差，年、月、日不清楚，可知上半年或下半年；只能单独在家附近行动，对现住地只知名称，不知道方位  7-9分：时间观念很差，年、月、日不清楚，可知上午或下午；只能在左邻右舍间串门，对现住地不知名称和方位  10分：无时间观念；不能单独外出 |
| b144 | Memory functions | Description: specific mental functions of registering and storing information and retrieving it as needed.  描述：登录和贮存信息并在需要时检索信息的特殊精神功能。 |
|  |  | Inclusions: functions of short-term and long-term memory, immediate, recent and remote memory; memory span; retrieval of memory; remembering; functions used in recalling and learning, such as in nominal, selective and dissociative amnesia  包括：短时和长时记忆、瞬时、近期和远期记忆功能；记忆跨度；记忆检索；记忆；用于回忆和学习的功能，如命名性、选择性和分离性遗忘症 |
|  |  | Exclusions: consciousness functions (b110); orientation functions (b114), intellectual functioning (b117), attention functions (b140), perceptual functions (b156); thought functions (b160), higher-level cognitive functions (b164), mental functions of language (b167); calculation functions (b172)  不包括：意识功能（b110）；定向功能（b114）；智力功能（b117）；注意力功能（b140）；知觉功能（b156）；思维功能（b160）；高水平认知功能（b164）；语言精神功能（b167）；计算功能（b172） |
|  |  | NRS assessment:  0: be able to maintain social, age-appropriate long- and short-term memory, can have a complete recall  1-3: mild memory disturbance or inability to recall  4-6: moderate memory disturbance or inability to recall  7-9: severe memory disturbance or inability to recall  10: complete memory disorders or a complete inability to recall past foods correctly  NRS评分：  0分，总是能够保持与社会、年龄所适应的长、短时记忆，能够完整的回忆  1-3分，出现轻度的记忆紊乱或回忆不能  4-6分，出现中度的记忆紊乱或回忆不能  7-9分，出现重度的记忆紊乱或回忆不能  10分，记忆完全紊乱或者完全不能对既往食物进行正确的回忆 |
| d710 | Basic interpersonal interactions | Description: interacting with people in a contextually and socially appropriate manner, such as by showing consideration and esteem when appropriate, or responding to the feelings of others.  Simple description: Interacting with people in a contextually and socially appropriate manner.  描述：以与社会背景适宜的方式于人交往，如在适当的时候表现出体谅和尊重，或者对别人的感觉做出反应。  简洁描述：以符合社会背景的恰当的方式与人交往。 |
|  |  | Inclusions: showing respect, warmth, appreciation, and tolerance in relationships; responding to criticism and social cues in relationships; and using appropriate physical contact in relationships  包括：在人际关系中表现出尊重、热情、感谢和宽容；对批评和社会暗示做出反应；采用适当的身体接触 |
|  |  | Exclusions: none  不包括：无 |
|  |  | NRS assessment:  0: be able to communicate with others normally  1-3: be able to express needs and understand what others saying, but it taking more time  4-6: difficulty in expressing or understanding, frequent repetition or simplification of oral expressions  7-9: serious difficulty in expressing or understanding, and needs a large amount of help from others  10: unable to express needs or understanding of others’ words, interpersonal communication is completely dependent  NRS评分：  0分：无困难，能与他人正常沟通和交流  1-3分：能够表达自己的需要及理解别人的话，但需要增加时间  4-6分：表达需要或理解有困难，需频繁重复或简化口头表达  7-9分：表达或理解有严重困难，需要大量他人帮助  10分：不能表达需要或理解他人的话,人际交往完全依赖 |
| b210 | Seeing functions | Description: sensory functions relating to sensing the presence of light and sensing the form, size, shape and colour of the visual stimuli.  描述：与感受存在的光线和感受视觉刺激形式、大小、形状和颜色有关的感觉功能。 |
|  |  | Inclusions: visual acuity functions; visual field functions; quality of vision; functions of sensing light and colour, visual acuity of distant and near vision, monocular and binocular vision; visual picture quality; impairments such as myopia, hypermetropia, astigmatism, hemianopia, colour-blindness, tunnel vision, central and peripheral scotoma, diplopia, night blindness and impaired adaptability to light  包括：视敏度功能；视野功能；视觉品质；感受光线和色彩、远近视敏度、单眼和双眼视觉功能；视图品质；如近视、远视、散光、偏盲、色盲、管状视、中心和周围盲点、复视、夜盲、光适应性损伤 |
|  |  | Exclusions: perceptual functions (b156)  不包括：知觉功能（b156） |
|  |  | NRS assessment:  0: can read standard typeface on books and newspapers  1-3: can read large type clearly, but can't read standard type on books and newspapers  4-6: limited vision and cannot read newspaper headlines, but can recognize objects  7-9: difficult to recognize objects, can only see light, color and shape  10: without eyesight, the eye cannot follow an object  NRS评分：  0分：能看清书报上的标准字体  1-3分：能看清楚大字体，但看不清书报上的标准字体  4-6分：视力有限，看不清报纸大标题，但能辨认物体  7-9分：辨认物体有困难，但眼睛能跟随物体移动，只能看到光、颜色和形状  10分：没有视力，眼睛不能跟随物体移动 |
| b230 | Hearing functions | Description: sensory functions relating to sensing the presence of sounds and discriminating the location, pitch, loudness and quality of sounds.  描述：与感受存在的声音和辨别方位、音调、音量和音质有关的感受功能。 |
|  |  | Inclusions: functions of hearing, auditory discrimination, localization of sound source, lateralization of sound, speech discrimination; impairments such as deafness, hearing impairment and hearing loss  包括：听、听觉辨别、声源定位、单侧声音、言语辨别的功能；如聋、听力损伤和听觉缺失的损伤 |
|  |  | Exclusions: perceptual functions (b156) and mental functions of language (b167)  不包括：知觉功能（b156）和语言精神功能（b167） |
|  |  | NRS assessment:  0: can have normal conversation, can hear TV, telephone and doorbell sound  1-3: cannot hear when speaking softly or at a distance of more than 2 meters  4-6: difficult to normal communicate, need to be in a quiet environment or speak loudly to hear  7-9: can only partially hear when speakers speak loudly or slowly  10: completely deaf  NRS评分：  0分：可正常交谈，能听到电视、电话、门铃的声音  1-3分：在轻声说话或说话距离超过2米时听不清  4-6分：正常交流有些困难，需在安静的环境或大声说话才能听到  7-9分：讲话者大声说话或说话很慢，才能部分听见  10分：完全听不见 |
